# Innovations in MALDI-TOF Mass Spectrometry: Bridging modern diagnostics and historical insights

**DOI:** 10.1515/biol-2025-1136

**Published:** 2025-07-18

**Authors:** Anna Andreadi, Evgenia Tsivelekidou, Iasonas Dermitzakis, Paschalis Theotokis, Sofia Gargani, Soultana Meditskou, Maria Eleni Manthou

**Affiliations:** Department of Histology and Embryology, Medical School, Faculty of Health Sciences, Aristotle University of Thessaloniki, Thessaloniki, 54621, Greece; 2nd Department of Neurology, University General Hospital AHEPA, Medical School, Aristotle University of Thessaloniki, Thessaloniki, Greece; School of Biology, Aristotle University of Thessaloniki, Thessaloniki, Greece

**Keywords:** MALDI-TOF MS, biomarkers, microbiology, paleopathology, prospects

## Abstract

Matrix-assisted laser desorption/ionization time-of-flight mass spectrometry (MALDI-TOF MS) is a robust analytical technology that has become integral to biomolecular research. Since its introduction into microbiology in the early 2000s, its versatility has enabled a wide spectrum of applications extending from routine microbial identification to advanced proteomic profiling, antimicrobial resistance testing, biomarker discovery, and even historical disease investigation. In proteomics, MALDI-TOF MS has proven valuable for identifying disease-associated proteins, with applications in oncology, metabolic disorders such as diabetes and dyslipidemia, neurodegenerative diseases, hemoglobinopathies, and neonatal screening. Additionally, it has facilitated pharmacokinetic studies by enabling detailed analysis of drug distribution and metabolism. Despite limitations such as dependency on reference databases and challenges in distinguishing closely related species, ongoing advancements continue to enhance its accuracy and range. The integration of MALDI-TOF MS with molecular methods like polymerase chain reaction further strengthens its diagnostic utility. This review aims to present recent technological progress while highlighting the expanding interdisciplinary utility of MALDI-TOF MS. Emphasis is placed on emerging fields, including paleopathology, where its potential remains underexploited. By outlining its evolving capabilities, we propose a conceptual framework that positions MALDI-TOF MS as a unifying platform capable of driving innovation across diverse scientific and biomedical disciplines.

## 1 Introduction

Over the past two decades, medical diagnostics has witnessed a transformative evolution marked by a growing demand for rapid, reliable, and cost-efficient tools capable of accurately identifying pathogens and biomarkers [1]. The increasing burden of infectious diseases, antimicrobial resistance, and complex multifactorial conditions such as cancer and neurodegeneration has heightened the need for sophisticated diagnostic modalities that can provide timely and actionable information. Traditional culture-based and biochemical identification methods, while historically foundational, are often slow, labor-intensive, and lack the sensitivity required for modern clinical decision-making [2]. Prior to the introduction of mass spectrometry (MS) into clinical workflows, diagnostics relied heavily on time-consuming processes that delayed treatment initiation and compromised patient outcomes [3]. This diagnostic gap underscored a pressing need for technological innovation – particularly in the form of analytical platforms that could offer high-throughput, precise molecular identification with minimal sample preparation [4].

MS, originally developed as a tool for fundamental analytical chemistry, has undergone significant transformation since its inception in the early 20th century [5]. Initial mass spectrometric technologies were primarily applied in the fields of physics and chemistry to measure atomic and molecular masses [6]. However, the landscape began to shift dramatically with the advent of biological applications in the latter half of the century [7]. A pivotal breakthrough came with the development of matrix-assisted laser desorption/ionization (MALDI) and its coupling with time-of-flight (TOF) analyzers, culminating in the matrix-assisted laser desorption/ionization time of flight mass spectrometry (MALDI-TOF MS) technique [8]. This innovation provided a soft ionization method suitable for the analysis of large biomolecules, including proteins and peptides, without fragmentation. MALDI-TOF MS rapidly gained traction due to its simplicity, speed, and suitability for biomolecular profiling [9]. The foundational work of Karas, Hillenkamp, and Tanaka – recognized with the 2002 Nobel Prize in Chemistry – was instrumental in establishing MALDI-TOF MS as a viable tool for life sciences [10]. Since then, the technique has evolved from a laboratory curiosity to a clinical cornerstone in microbial identification and beyond.

Recent years have witnessed a cascade of innovations that have elevated MALDI-TOF MS beyond its initial microbial identification capabilities. Technological advancements have improved sensitivity and resolution, enabling the detection of subtle biomolecular differences and the quantification of post-translational modifications [11]. Data processing has also evolved, with artificial intelligence (AI) and machine learning algorithms now integrated into spectral analysis workflows, enhancing classification accuracy and predictive capacity [12]. MALDI-TOF MS has expanded its utility across diverse biomedical domains, including proteomics, metabolomics, biomarker discovery, oncology, pharmacokinetics, and even environmental microbiology [13]. Its transition from a single-use diagnostic tool to a multipurpose analytical platform exemplifies its growing significance. Furthermore, its role in detecting antimicrobial resistance, characterizing complex protein profiles, and enabling non-invasive liquid biopsies underscores its value in precision medicine [14].

Beyond its contemporary clinical applications, MALDI-TOF MS also embodies a fascinating confluence of historical and modern science. The technology’s core principles are deeply rooted in the heritage of analytical chemistry, where instrument-based identification has long guided scientific understanding [5]. Today, those foundational techniques have matured into robust diagnostic strategies that bridge centuries of knowledge. MALDI-TOF MS illustrates this continuity by offering tools that are simultaneously informed by traditional mass spectrometric theory and enabled by modern biomedical imperatives [15]. Moreover, its emerging role in paleopathology – facilitating the detection of ancient biomarkers and pathogens – highlights the method’s capacity to unify historical inquiry with present-day technological prowess [16]. In doing so, it strengthens our understanding of disease evolution and fosters an interdisciplinary dialogue between clinical diagnostics and archaeological science.

This review aims not only to present recent technological advances in MALDI-TOF MS but also to illuminate its expanding utility across a wide range of biomedical and interdisciplinary fields. Moving beyond its established diagnostic roles, we highlight how MALDI-TOF MS is being positioned as a versatile analytical platform with transformative potential – even in areas such as paleopathology, where its application has been largely underexplored. By tracing the evolution of its capabilities and demonstrating its relevance across both contemporary and historical biomedical contexts, this review underscores MALDI-TOF MS as a unifying tool that can bridge diverse scientific disciplines. Through this lens, we encourage researchers and clinicians to consider its applicability not only within conventional boundaries but also in uncharted scientific territories where molecular analysis may provide critical insights. In doing so, we propose a broadened conceptual framework for how MALDI-TOF MS can inform future discovery and cross-disciplinary integration.

## 2 Review methodology

This review, although not systematic, follows specific methodology. A meticulous research was conducted in databases like PubMed, Scopus, and Web of Science, using relevant keywords such as “MALDI-TOF MS,” “MALDI-TOF MS in paleopathology,” “MALDI-TOF MS in medicine,” “MALDI-TOF MS in microbiology,” “MALDI-TOF MS biomarkers,” and other related. In addition, the reference list of identified articles was examined for further sources. Inclusion criteria involve articles with full-text availability. The title and abstracts of the articles were assessed. Although formal quality appraisal tools were not employed, all relevant limitations, such as confounding bias, were acknowledged for transparency reasons. The key findings of the included articles are summarized to showcase in a narrative way the capability and potential MALDI-TOF MS in medical and non-medical fields.

## 3 MALDI-TOF MS operation principles

MALDI-TOF MS is a powerful analytical technique extensively employed in proteomics, microbiology, and clinical diagnostics for the rapid identification and characterization of biomolecules. The technique combines soft ionization with high-resolution mass analysis, enabling the detection of high-molecular-weight, fragile molecules such as proteins, peptides, and polymers while minimizing fragmentation [17]. The method relies on the use of a crystalline matrix that absorbs laser energy to facilitate the ionization of the analyte, which is then analyzed based on the time it takes to accelerate through a flight tube under vacuum [17]. In more detail, bacterial detection using MALDI-TOF MS proceeds as follows: some bacterial samples, like Gram-positive bacteria, need to be prepared before extraction [18]. On the other hand, Gram-negative bacteria can be identified directly by MS, a technique called direct cell profiling [19]. During MALDI-TOF MS analysis, samples are mixed or coated with an energy-absorbent matrix solution that ensnares and co-crystalizes them when dried [20]. When the matrix is irradiated with a laser beam (typically a nitrogen laser at a wavelength of 337 nm or a neodymium-doped yttrium-aluminum-garnet laser [Nd:YAG laser] at a wavelength of 355 nm), it absorbs its energy, generating ions that charge the analytes in the sample [20].

Matrix is an organic molecule that can absorb radiation and scatter gas molecules effectively. Common matrices include 2,5-dihydroxybenzoic acid, α-cyano-4-hydroxy-*trans*-cinnamic acid, and sinapinic acid [17]. The ions produced are accelerated by an electric field based on their mass-to-charge ratio (*m*/*z*) and are measured by the TOF mass analyzer. When a TOF fails to detect the target analytes in a complex mixture, a second TOF is incorporated, so that tandem mass analysis is performed [17]. A typical mass range *m*/*z* of 2–20 kDa is used, which corresponds to ribosomal and gatekeeping proteins. After TOF analysis, the peptide mass fingerprint (PMF) of the sample is produced and the PMF of an unknown organism or biomarker is compared to known database PMFs [19]. Those databases are provided by the Bruker and the Shimadzu systems and are expanding along with the machine’s increased use providing even more specific results [21]. Due to its speed, sensitivity, and minimal sample preparation requirements, MALDI-TOF MS has become an indispensable tool in both research and clinical laboratories.

## 4 Conventional applications of MALDI-TOF MS in medical sciences

**Figure 1:**
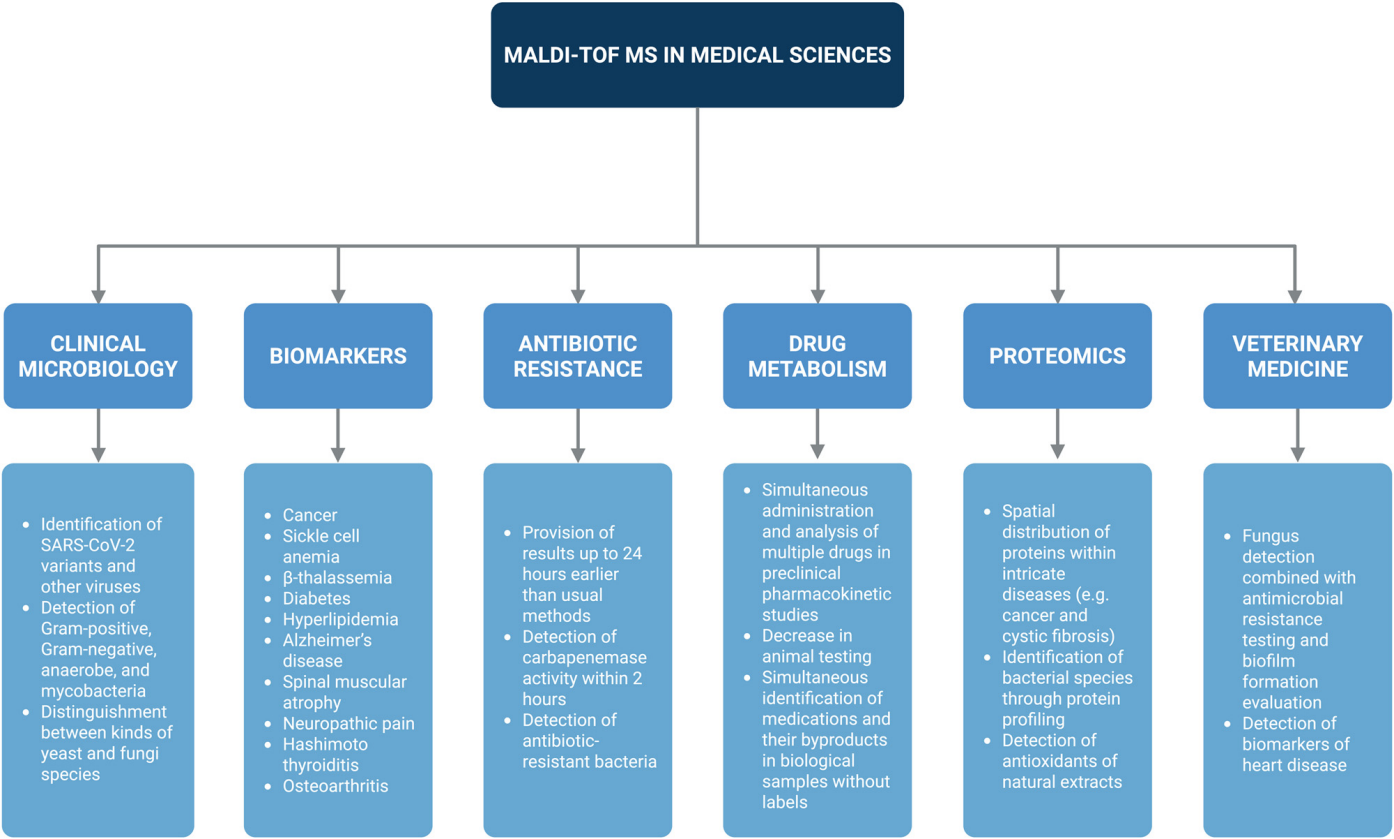
Conventional applications of MALDI-TOF MS in medical sciences.

MALDI-TOF MS has emerged as a powerful analytical platform with remarkably diverse applications across the medical sciences. Its versatility lies not only in its ability to deliver rapid and highly accurate molecular identification, but also in its adaptability to a wide range of clinical contexts – from pathogen detection and antimicrobial resistance monitoring to biomarker discovery, drug metabolism studies, and even veterinary diagnostics (Figure 1). This broad utility underscores its potential to replace or complement traditional diagnostic methods, improve patient outcomes through faster clinical decision-making, and expand precision medicine approaches. The following sections aim to demonstrate how the wide-ranging applications of MALDI-TOF MS are redefining standards in modern healthcare by providing robust, scalable, and cost-effective solutions across multiple disciplines.

### 4.1 Clinical microbiology

#### 4.1.1 Identification of bacteria

One of the strengths of MALDI-TOF MS is its ability to work well with a range of different types of microbes that are usually challenging to detect using traditional methods. It has been very successful in identifying many types of bacteria: Gram-positive, Gram-negative, anaerobe, and mycobacteria, as well as in fungi species, too. MALDI-TOF MS was used to distinguish *Lactobacillus plantarum* by observing 34 protein markers [22]. Similarly, it has proven to be highly accurate in differentiating between related strains of clinical *Streptococci*, with researchers successfully identifying 99 distinct species through this approach [23]. The detection of *tuberculosis* through mycobacteria analysis depends heavily on MALDI-TOF MS because it can identify *Mycobacterium tuberculosis* [24]. The technique has also demonstrated a remarkable 95.7% accuracy in identifying anaerobic bacteria, which are often difficult to cultivate [25].

Furthermore, MALDI-TOF MS has played a role in differentiating Gram-negative bacterial rods like those belonging to the Enterobacteriaceae group, including *Escherichia coli*. Research conducted by Almuzara et al. demonstrated the system’s precision in recognizing species obtained from patients with cystic fibrosis [26]. Additionally, *Acinetobacter* species have been accurately identified through the utilization of expanded databases [27]. In the field of microbiology, this approach has proven to be very reliable in distinguishing between species of *Staphylococci*, making traditional coagulase tests unnecessary. Carbonelle et al. found a 97% accuracy rate in identifying coagulase-negative *Staphylococci* (CoNS) using the Bruker BioTyper database [28]. Meanwhile, Spanu et al. achieved a 99% precision rate when identifying subspecies, highlighting its cost-effectiveness compared to DNA sequencing [29]. While some inconsistencies still exist, improvements in reference databases and spectral matching algorithms are gradually addressing these issues.

The clinical microbiology field has experienced a transformation through MALDI-TOF MS as this technology provides rapid and precise pathogen identification across different uses. The technique allows fast bacterial pathogen detection in periprosthetic joint infections (PJIs) achieved in hours instead of the typical days required by traditional culture methods. The sensitivity of the technique improves through sonication methods, which remove bacteria from biofilm-covered prosthetic implants, thus increasing detection success rates [30]. The MALDI-TOF MS assays for alpha-defensin in synovial fluid provide a fast diagnostic solution for PJIs because they produce results in about 20 min, which helps doctors make intraoperative treatment choices.

The application of MALDI-TOF MS extends beyond orthopedic uses because it helps identify both uncommon and difficult-to-culture pathogens. The identification of *Helicobacter cinaedi* in immunocompromised patients becomes possible through MALDI-TOF MS which enables immediate antimicrobial therapy. The technique allows researchers to distinguish gastric *Helicobacter* species through their distinct protein patterns leading to better diagnostic accuracy [31]. The combination of cost-effectiveness and high-throughput capabilities establishes MALDI-TOF MS as an ideal tool for environmental monitoring within cleanroom settings to identify *Bacillus* species and related microbes. The system’s quick processing features enable its use in regular surveillance operations which maintain contamination control in critical environments [32]. The wide range of applications and high efficiency of MALDI-TOF MS demonstrates its essential position in present-day diagnostic procedures.

#### 4.1.2 Identification of COVID-19 and other viruses

When it comes to virology, in 2019 the world faced a health crisis with the emergence of coronavirus disease of 2019 (COVID-19) caused by the virus called severe acute respiratory syndrome coronavirus 2 (SARS-CoV-2). Initially, doctors used methods like polymerase chain reaction (PCR) for the diagnosis but in 2020, Nachtigall et al. came up with a method using MALDI-TOF MS analysis of nasal swabs, which showed an impressive accuracy of 93% in identifying cases from a sample size of 362, including both positive and negative cases [33]. Yan et al. extended their research by using MALDI-TOF MS to examine blood samples from individuals with COVID‐19 symptoms compared to COVID-19 patients and healthy individuals [34]. They developed a model that demonstrated 99% accuracy along with 98% sensitivity and 100% specificity. Additionally, in 2020, Wang et al. applied PCR-MALDI-TOF MS to detect SARS-CoV-2 nucleic acids. In their study, sputum and pharyngeal swab samples were analyzed for two specific genes [35]. The study revealed that SARS-CoV-2 detection by PCR-MALDI-TOF MS had high accuracy, sensitivity, and specificity and could be used in clinical settings to improve SARS-CoV-2 nucleic acids testing efficiency. At the end of 2021, Zhao et al. developed a novel strategy of a MALDI-TOF-based multiplex PCR mini-sequencing technique to identify SARS-CoV-2 variants [36]. Nine mutant types of SARS-CoV-2 variants were detected, and high specificity and an accuracy of 100% were achieved among 20 clinical verification samples. In 2022, Han et al. reported a Y-structure-induced rolling loop amplification method combined with MALDI-TOF MS for nucleic acid detection of SARS-CoV-2 with high specificity and velocity [37]. Compared with the real-time PCR test, which was the gold standard for SARS-CoV-2 early diagnosis approved by the World Health Organization (WHO), the PCR-MALDI-TOF MS-based assay had superior performance in the discrimination of SARS-CoV-2 variants [17,38].

MALDI-TOF MS was not considered the diagnostic gold standard of COVID-19. MALDI-TOF MS has shown promise in detecting SARS-CoV-2, but studies have indicated that its sensitivity may not consistently match that of reverse transcription polymerase chain reaction (RT-PCR). For example, an exploratory study using MALDI-TOF MS on saliva samples achieved an accuracy of 85.2%, which, although significant, may not be sufficient for primary diagnostic purposes [39]. In addition, implementing MALDI-TOF MS for viral detection would require significant investment in equipment and training. Given the urgency of the pandemic, using existing RT-PCR infrastructure was more feasible for timely mass testing. In addition, MALDI-TOF MS was used for detecting various viruses like polioviruses and respiratory viruses which have indicated its potential as a supporting tool for traditional virological diagnostics [7]. Recent investigations have also delved into the integration of MS with machine learning to improve the speed and cost-effectiveness of diagnosing infections. The outcomes have shown promising advancements in accuracy, following model refinement [40]. MALDI-TOF MS has proven to be successful in distinguishing viruses, like herpes simplex virus and human papillomavirus accurately in clinical virology. This method has also been effective in differentiating hepatitis B and C viruses along with influenza viruses and human immunodeficiency virus [41].

#### 4.1.3 Identification of fungi

MALDI-TOF MS has also shown its effectiveness in identifying fungi, like yeasts and molds in environments successfully. This method allows precise identification of fungal types at a reasonable cost by comparing the mass spectra of unknown samples with established databases containing known fungal spectra. Since its incorporation into the field of mycology, MALDI-TOF MS has greatly enhanced the capability to distinguish closely related yeast species like those found in the *Candida* complex. These species are often challenging to differentiate using biochemical techniques [42]. Numerous research studies have emphasized the effectiveness of MALDI-TOF MS in identifying both yeasts and filamentous fungi. However, having access to databases is crucial for this identification process [43]. Recent progress has also enabled the use of MALDI-TOF MS in identifying filamentous fungi like *dermatophytes*, which usually present obstacles due to their intricate cell walls and varied shapes [43]. Through enhancements to the databases utilized by MALDI-TOF MS studies, MALDI-TOF MS has become a valuable tool for precisely recognizing various fungal infections [42].

MALDI-TOF MS provides an affordable option for identifying bacteria and fungi compared to the lengthy and costly process of broad-range PCR followed by sequencing. The use of MALDI-TOF MS allows for the identification of colonies in just a few minutes without the need to determine beforehand if the sample is bacterial or fungal, making it an effective method for clinical applications [44]. However, a notable drawback of MALDI-TOF MS is its requirement for pre-treatment of the sample, especially when dealing with fungi, molds, and *Mycobacteria*. This step not only consumes time but also calls for experienced staff and inflates the overall expenses of the diagnostic procedure. The effectiveness of MALDI-TOF MS in this context remains unclear and thus it is not commonly used in labs for cases like an athlete’s foot [45].

Nonetheless, MALDI-TOF MS has proven to be very effective in distinguishing between kinds of yeast and fungi species, as researchers found that 96% of *Candida* isolates from 15 different species were correctly identified using this method [46]. Moreover, two recent studies have also reported accuracy rates in yeast identification through MALDI-TOF MS, since it outperforms other conventional phenotypic methods of identifying yeast organisms by accurately telling apart various species like *Candida dubliniensis* and *albicans* as well as *Cryptococcus neoformans* and *gattii* along with other *Candida* complexes [44,47,48]. However, there is still little data available on how well it can differentiate molds like *Aspergillus*, *Penicillium*, *Fusarium*, and *dermatophytes*, as it was in 2017 when the first global database of fungi was created, which contained over 11,000 reference spectra from 938 fungal species, such as *Aspergillus*, *Trichophyton*, or *Microsporum* [49].

### 4.2 Establishing biomarkers

Cancer diagnosis and prognosis account for a large proportion of MALDI-TOF MS-based clinical disease diagnoses (Table 1). For example, many studies have reported the use of MALDI-TOF MS in distinguishing ovarian cancer from healthy controls. Cancer antigen 125 (CA125) is one of the two biomarkers for the diagnosis of recurrence and treatment response in ovarian cancer [17]. CA125 alone can predict ovarian cancer up to 9 months before diagnosis; however, it is not a specific cancer antigen, and it might be regulated by other benign gynecological diseases as well. Periyasamy et al. suggested that solid-phase extraction before MALDI-TOF MS analysis can improve the sensitivity of a diagnosis model to differentiate serous adenocarcinoma (a common type of epithelial ovarian cancer) and healthy controls [50]. Swiatly et al. proposed that the combined usage of isobaric tags for relative and absolute quantification (iTRAQ)-based quantitative proteomic analysis and MALDI-TOF MS can improve the differentiation of benign and malignant tumors in ovarian cancer [51].

**Table 1:** Diagnostic applications of MALDI-TOF MS

Cancer type	Application of MALDI-TOF MS	References
Ovarian cancer	Distinguishing OC from healthy controls	[17,50,51]
Prostate cancer	Early diagnosis and biomarker discovery	[17,22]
Multiple myeloma	Diagnosis through Bence-Jones protein detection in urine and screening by machine learning	[52,53]
Melanoma	Biomarker discovery using exosomes and predicting progression	[17,58]
Osteosarcoma	Differentiating with/without lung metastasis	[37]
Breast cancer	Detection of BRC1 and BRC2 genes	[5,54]
Lung cancer	DNA methylation as a biomarker and other biomarkers	[17,59]
Colorectal cancer	Analyzation of UTP23 protein	[57]

The MALDI-TOF MS technique has been used for the early diagnosis and prognosis of many other cancers including prostate cancer (PCa), liver cancer, multiple myeloma, breast cancer, pancreatic cancer, osteosarcoma, lung cancer, and colorectal cancer, too. In 2019, Long et al. proposed a MALDI-TOF MS-based approach for the diagnosis of multiple myeloma by detecting Bence-Jones proteins in human urine samples [52]. Combined with machine learning, it can be a useful screening tool of the disease [53]. In 2020, Sun et al. applied the technology of MALDI-TOF MS serum peptide fingerprinting to pre-diagnose PCa [22]. Recently, MALDI-TOF MS fingerprinting was further applied to discover lipid PCa biomarkers in urine samples from 121 PCa patients and 18 healthy controls [17]. Scientists have also achieved the identification of breast cancer gene 1 (BRC1) and breast cancer gene 2 (BRC2) copies through MALDI-TOF MS in 2023 which demonstrated better sensitivity than targeted next-generation sequencing at equivalent cost [54]. The exosome fingerprinting method using MALDI-TOF MS showed excellent sensitivity for pancreatic cancer detection which indicates its potential as a monitoring tool for this cancer type [55]. The same technique has also been applied to the diagnosis of osteosarcoma using non-invasive liquid biopsy [37]. Li et al. used MALDI-TOF MS to analyze serum samples and proposed multiple candidates for lung cancer biomarkers to evaluate chemotherapy effectiveness [56]. Researchers have also used MALDI-TOF MS to analyze UTP23 protein which promotes colorectal cancer progression thus demonstrating its value in cancer research [57].

On the mature foundation of MALDI-TOF MS fingerprinting technology, many other samples have been used for biomarker discovery and cancer diagnosis, especially exosomes isolated from body fluids. In 2019, Zhu et al. used MALDI-TOF MS to analyze exosomes extracted from the serum of melanoma patients and healthy donors and demonstrated that the mass fingerprinting of bloodstream-circulating exosomes can be used for cancer diagnosis and monitoring [58]. In 2021, Han et al. successfully used MALDI-TOF MS to analyze serum exosomes from patients with osteosarcoma with and without lung metastasis and healthy controls [37]. In 2021, cells collected from the surface of suspected skin areas were used by Zhu et al. to detect melanoma and predict skin disorder progression [58]. Non-invasive sampling is another advantage of this study considering the benign method of obtaining cells from the skin surface [17]. DNA methylation is another potential biomarker for several cancers with close relatedness to tumorigenesis, development, and cell carcinogenesis. Ehrich et al. used the method to quantify methylation differences between normal and neoplastic lung cancer tissue samples [59].

Furthermore, MALDI-TOF MS can potentially be used for neonatal screening for various diseases, such as phenylketonuria, homocystinuria, maple syrup urine disease, and sickle cell anemia. Kim et al. found that parylene matrix chips in combination with MALDI-TOF MS are an approach to testing babies for metabolic conditions, such as phenylketonuria and maple syrup urine disease [60]. Despite some challenges in the extracting efficiency encountered in this process, the technique demonstrates consistency and accuracy levels rendering it a valuable asset for precise diagnosis in medical facilities [60]. In a pilot screening study for sickle cell anemia that took place in France, researchers managed to differentiate between heterozygous fetal hemoglobin (F), sickle hemoglobin (S), and hemoglobin E (FSE), fetal hemoglobin (F), sickle hemoglobin (S), and hemoglobin O-Arab (FSO-Arab) and β-thalassemia trait (S-β+) samples and heterozygous fetal hemoglobin, adult hemoglobin, and sickle cell hemoglobin (FAS) and homozygous fetal hemoglobin (F) and hemoglobin S (FS) samples [61]. Furthermore, a prospective study in 2024 concluded that the sensitivity and specificity of the method reached 100% [62]. The MALDI-TOF MS system allows the detection of fetal hemoglobin (Hb-F) variants and exact diagnosis and classification of β-thalassemia which assists in personalized treatment of β-thalassemia [63]. Furthermore, researchers have developed an MS/MS Hemo kit for the detection of sickle cell anemia with almost 100% sensitivity and specificity [64]. MALDI-TOF MS was proven to be a cost- and time-effective asset that enabled its approval for screening populations [61].

Biomarkers for diabetes mellitus are also targets of MALDI-TOF MS. Meng et al. identified six peptides linked to diabetes mellitus type 2 that can differentiate patients from healthy controls with an accuracy of 82.20%, a sensitivity of 82.50%, and a specificity of 77.80% [65]. The distinction between type 1 and type 2 diabetes can also be achieved, as MALDI-TOF MS can detect the quantities of C-reactive protein, which represents the amount of insulin in the body [66]. In 2021, Zawada et al. attempted to further investigate the protein profile of type 1 diabetes patients with certain characteristics (increased adipose tissue, decreased control of the disease, and chronic complications) and concluded that increased levels of C3, C4, and fibrinogen should be taken into consideration when it comes to the disease [67]. Recent studies have shown that MALDI-TOF MS technology shows promise for individualized diabetes treatment and diabetic microangiopathy genetic factor investigation [68]. MALDI-TOF MS has been proven useful in recent studies for detecting protein-related molecules linked to hyperlipidemia, which demonstrates its potential as a biomarker for disease diagnosis and research [69].

Alzheimer’s disease (AD) is a neurodegenerative disease and in the early phase of its discovery, AD biomarkers, cerebrospinal fluid (CSF), a proximal fluid, was often studied. According to a review, the most characteristic AD biomarkers in CSF are β-amyloid (βA), tau protein, and phospho-tau [70]. In 1993, the first study of targeted βA proteomics found multiple βA isoforms in CSF with the use of MALDI-TOF MS [71]. In 2018, Nakamura et al. found a new composite biomarker, βA precursor protein (APP), to predict positive or negative brain βA based on MALDI-TOF MS analysis, illustrating the high performance of plasma biomarkers in brain βA burden prediction and AD diagnosis [72]. In 2021, Shimadzu released a MALDI-based amyloid mass spectrometry with a centiloid system (amyloid MS CL system) for testing the levels of amyloid peptides in the blood that are associated with AD [17]. The application of MALDI-TOF MS as a screening tool for spinal muscular atrophy and for identifying biomarkers of neuropathic pain has been proposed [73,74]. MALDI-TOF MS is nowadays capable of identifying novel biomarkers for Hashimoto thyreoiditis and osteoarthritis, too [75,76].

### 4.3 Detection of antibiotic resistance

MALDI-TOF MS has proven effective in detecting antibiotic resistance. Its quick and accurate detection abilities offer information for care and community health measures. MALDI-TOF MS plays a role in enhancing antimicrobial susceptibility testing by swiftly detecting resistance mechanisms compared to the conventional methods – providing results up to 24 h earlier than usual methods would offer. This technology can pinpoint resistance through approaches such as analyzing compounds and examining antimicrobial molecules, changes in bacterial cell wall, ribosomal RNA methylation, and mutations. Its applications are diverse, including detection of carbapenemase activity within 2 h and identifying colistin resistance by studying lipid A biomarkers [7]. Some of the accomplishments of this method include targeting B lactamases and methicillin-resistant *Staphylococcus aureus* in addition to the carbapenemase gene (cfiA gene) in *Bacteroides fragilis* and vancomycin-resistant *Enterococcus* [77,78,79]. In 2018, Zhu and colleagues altered the material used in MALDI-TOF MS testing to analyze extended-spectrum β-lactamase-producing *E. coli*, multidrug-resistant *Pseudomonas aeruginosa*, and methicillin-resistant *S. aureus* using intact bacteria [58].

### 4.4 Study of drug metabolism

MALDI-TOF MS is a valuable tool in research that aids accurately and sensitively in examining drug substances and their byproducts in biological samples and in comprehending the processes related to absorption, distribution, metabolism, excretion, and toxicity. Its use has become widespread for examining how drugs are distributed within tissues without the requirement of labeling them specifically. This helps localize and measure drug components accurately with resolution in pharmacokinetic investigations [80,81]. This method enables scientists to chart the spread of drugs and their byproducts in tissues and gain insight into the effects and potential dangers of the medication [80].

Additionally, Swales et al. demonstrated the utility of MALDI-TOF MS in cassette dosing strategies, which enable the simultaneous administration and analysis of multiple drugs in preclinical pharmacokinetic studies [81]. This method decreases animal testing while boosting biodistribution analysis throughput which leads to more efficient drug discovery processes. MALDI-TOF MS works together with drug efficacy study implementation and liquid extraction surface analysis MS imaging techniques to provide complete drug distribution detection and analysis. Herkt et al. utilized MALDI-TOF MS for the pharmacokinetic study of antisense oligonucleotides [82]. The technique enabled the identification and measurement of therapeutic oligonucleotides in biological samples with a focus on plasma analysis. This is crucial for the advancement of treatments that target materials like antisense oligonucleotides for ailments such as cardiac hypertrophy. MALDI-TOF MS is also utilized for examining how drugs bind to proteins and analyzing metabolites in pharmacokinetics. This contributes to the identification of drug–protein connections, the monitoring of drug stability, and the interactions with molecules during the distribution and metabolism stages of pharmacokinetics [82].

In general, MALDI-TOF MS greatly improves the accuracy of research by delivering detailed and simultaneous identification of medications and their byproducts in biological samples without labels. This aids in improving comprehension of how drugs act in the body, resulting in informed choices throughout the drug development. The antioxidant and anticancer effects of natural extracts, such as those from *Verbascum thapsus*, have been explored using MALDI-TOF MS, further demonstrating its relevance in pharmacological studies and reinforcing the technique’s capability to analyze complex biological samples and contribute to drug development [83]. Additionally, recent researches in herbal medicines have utilized MALDI-TOF MS to illustrate signaling pathways related to drug action [84].

### 4.5 Proteomics

MALDI-TOF MS has undergone advancements in its use for proteomics over the years. Initially, in 2006, it became a tool for quickly identifying proteins in large quantities [85]. By 2007, advancements in high-performance systems improved its sensitivity and speed, making it a popular choice for studying proteins and peptides in proteomic research, and in 2011, bacterial species were identified through protein profiling [86]. This method became a practice in microbial diagnostics leading to 2015 when these advancements were further expanded to benefit the health field by aiding in the identification of bacteria and enhancing the understanding of antimicrobial resistance [85,87]. In 2016, and beyond that time frame, especially in 2018 and 2022, MALDI-TOF MS technology made progress in exploring proteomics with a specific focus in mind, incorporating MALDI Fourier transform ion cyclotron resonance for an in-depth view of proteins in tissues to facilitate studies into the spatial distribution of proteins within intricate diseases, like cancer and cystic fibrosis [87]. Further enhancements occurred when the method was employed for pathogen identification and biomarker discovery purposes. This involved analyzing proteins and utilizing bioinformatics for more precise diagnostics [85,86]. In conclusion, MALDI-TOF MS has made significant contributions in the field of proteomics, ranging from basic protein identification to innovative uses in spatial proteomics and the discovery of pathogen biomarkers.

### 4.6 Veterinary medicine

Veterinary medicine also recognizes MALDI-TOF MS as a valuable diagnostic tool. The analysis of fungus detection combined with antimicrobial resistance testing and biofilm formation evaluation and heart disease biomarkers can be detected using MALDI-TOF MS [88,89,90]. The combination of MALDI-TOF MS with human pathogen databases through web applications enables precise detection of animal fungal infections [91]. The application of human pathogen databases in veterinary medicine demonstrates both the necessity and capability for interdisciplinary MALDI-TOF MS use to expand its functionality beyond current database constraints.

## 5 Expanding the application horizons of MALDI-TOF MS: A prism into paleopathology

**Figure 2:**
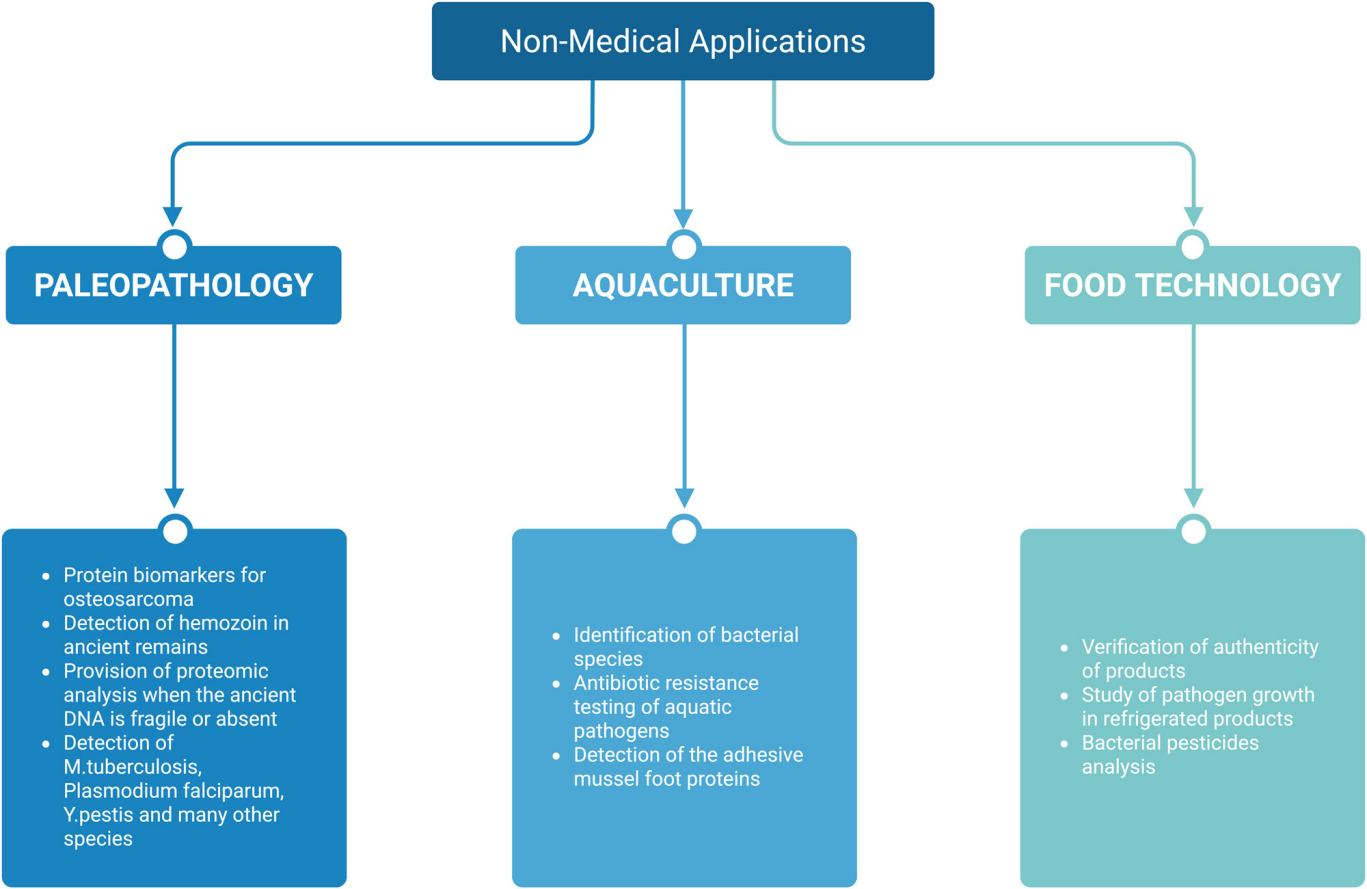
Non-medical applications of MALDI-TOF MS.

MALDI-TOF MS has transitioned from an experimental concept to a practical application in paleopathology (Table 2), (Figure 2). MALDI-TOF MS provides proteomic analysis through its stable molecular framework because PCR-based methods depend on the fragile and often degraded nucleic acids [17]. The method works best in extreme environmental settings because it recovers DNA when other methods fail [92].

**Table 2:** Summary of palaeopathological research with MALDI-TOF MS

Samples	Trait identified	References
Archaeological bone samples	*Mycobacterium tuberculosis*	[93]
Morphologically “healthy” human skeletal remains	*Mycobacterium tuberculosis*	[94]
Skeleton that presented evidence of tuberculous spondylitis in the Roman Period	*Mycobacterium tuberculosis*	[95]
Ancient, fragmented skeleton with osteogenic sarcoma/various infected and morphologically “healthy” human skeletal remains	Osteosarcoma and *Mycobacterium tuberculosis*	[102]
Coprolite in a closed Medieval barrel	15 different bacterial species of intestinal flora	[101]
Skeletal remains from indigenous Caribbean people	*Plasmodium falciparum*	[98]

In 2010, MALDI-TOF MS was used in a study to detect the presence of ancient mycolic acids from archaeological bone samples. It appeared to be a high-throughput and highly sensitive method for the molecular analysis of paleopathological remains infected by the *M. tuberculosis*. This was the first time that MALDI-TOF MS was demonstrated as a useful technique in the field of paleoanthropological investigations [93].

In 2011, the first study to show that the MS-based protein analysis of ancient proteins is a powerful technique for paleopathological examinations was executed with the identification of ancient mycobacterial proteins on infected and morphologically “healthy” human skeletal remains. Several mycobacterial proteins have been extracted and identified by MALDI-TOF MS from archaeological human skeletal remains since then [94,95]. It is worth mentioning, too, that given previous studies, MALDI-TOF MS not only successfully identifies ancient mycolic acids and proteins but also distinguishes *M. tuberculosis* from *Mycobacterium leprae*. According to the researchers, even though it has not been used in ancient bones, it is time to examine bones with evidence of leprosy and positive ancient DNA with the use of MALDI-TOF MS [96].

In 2023, *Plasmodium falciparum* was successfully identified in contemporary blood samples, facilitating research for other subspecies on a clinical basis [97]. However, beforehand, in 2017, Inwood J. had already written a thesis intending to create a method for the detection of *P. falciparum* infection [98]. A combination of three techniques was used: electron microscopy, X-ray diffraction, and MALDI-TOF MS and for the first time, detection of hemozoin in ancient remains was carried out. Hemozoin is a biomolecule produced by all *Plasmodium* species and is a promising biomarker for the identification of malaria both in living blood samples and ancient skeletal remains [98]. Although MALDI-TOF MS has not been used yet for the detection of *Yersinia pestis* in paleopathological findings, it has nevertheless been successfully used in modern clinical samples [68,99]. Antibodies of *Y. pestis* have been detected in ancient dental pulp using immunological techniques instead [100]. A coprolite found in 1996 inside a closed medieval barrel in the Namur region of Belgium underwent a multi-level analysis which included microscopy, culture, MALDI-TOF MS identification of colonies, and metagenomic analysis to determine the intestinal microbiome. The MALDI-TOF MS identification revealed 15 different bacterial species [101]. The MALDI-TOF MS use expanded successfully in 2014 from contemporary samples to the identification of protein biomarkers for osteosarcoma from a 2,000-year-old female skeleton excavated from Hungary. Up to 60 proteins were detected, among which many related to oncogenesis. The same study also managed to identify protein biomarkers of *M. tuberculosis* [102].

## 6 Translating the applications of MALDI-TOF MS beyond medicine

The aquaculture industry has adopted MALDI-TOF MS as a powerful identification tool as it provides faster results at lower costs than traditional bacterial identification techniques. The identification process of *V. anguillarum* together with rainbow trout pathogens *Aeromonas salmonicida*, *Flavobacterium psychrophilum*, *Yersinia ruckeri*, *Streptococcus agalactiae*, *Streptococcus iniae*, *Aeromonas hydrophila*, and *Aeromonas veronii* can be achieved through an efficient and cost-effective alternative method using MALDI-TOF MS [24,25,57,58]. MALDI-TOF MS has gained widespread acceptance in species-level identification and antibiotic resistance testing of aquatic pathogens during the last 10 years [103]. Indeed, with the use of MALDI-TOF MS, mussel foot proteins with strong adhesive properties were identified [104]! The implementation of MALDI-TOF MS technology has brought substantial improvements to food quality control and safety assessment procedures as well. The detection system based on MALDI-TOF MS has been implemented to verify Mozzarella di Bufala Campana cheese authenticity while protecting consumer safety [105]. The analysis of broiler chicken breast meat through MALDI-TOF MS during refrigerated storage revealed the progressive growth of *Pseudomonas* species numbers throughout storage time [106]. Additionally, MALDI-TOF MS has assisted in bacterial pesticide analysis to study their operational mechanisms and evaluate their potential to contaminate food products and detect remaining residues [107].

## 7 Future directions and current limitations of MALDI-TOF MS

In recent years, advances in MS have transformed microbial diagnostics, with MALDI-TOF MS emerging as a compelling alternative to traditional molecular methods. Specifically, MALDI-TOF MS offers an alternative to PCR, a standard method in today’s clinical and research praxis, with the advantages of speediness, cost efficiency, and ease of sample preparation [108]. Regarding the identification of substances or microorganisms in clinical scenarios, MALDI-TOF MS has proven valuable for making quick decisions about patient care needs [109], since it can pinpoint an isolate in 6 min while PCR usually requires more time due to its amplification steps and potential sequencing requirements. Moreover, MALDI-TOF MS is a budget-friendly option as it can cut costs by up to 32% in contrast to traditional techniques because of its inexpensive supplies and simplified procedure, particularly suited for extensive clinical microbiology labs, unlike PCR, which demands expensive materials and machinery [44]. MALDI-TOF MS has also benefits when it comes to preparing samples because it can pinpoint pathogens directly from samples without the requirement of culturing them first [18,44]. This proves to be particularly advantageous not only for diagnostics but for reducing sample damage, as well, especially when dealing with precious ancient samples. While PCR outshines in detecting hard-to-culture organisms and spotting resistance genes, MALDI-TOF MS is progressing in this realm by detecting proteins linked to resistance-like strains that produce carbapenemase [14]. MALDI-TOF MS stands out as a choice for quick and budget-friendly identification of microbes compared to PCR, which shines in genetic analysis and detecting resistance factors [44,110].

Beyond microbial identification, MALDI-TOF MS is gaining traction as a multifaceted tool in both clinical and research environments, offering applications that extend well into antimicrobial stewardship and disease biomarker discovery. Interestingly, MALDI-TOF MS generally provides precise microorganism identification that outperforms conventional biochemical and molecular techniques in terms of speed, cost-effectiveness, and convenience [108]. It plays a key role in combating the escalating threat of antimicrobial resistance by swiftly pinpointing antibiotic resistance and identifying different microbial species with accuracy and efficiency in crucial situations [111]. Additionally, the ability of MALDI-TOF MS to support susceptibility testing by identifying resistance mechanisms early on compared to traditional approaches highlights its crucial role in combating resistant infections [112]. In the field of proteomics, MALDI-TOF MS has made progress in identifying proteins linked to diseases like cancer and diabetes. This advancement has improved our knowledge of disease processes and opened up possibilities for personalized medical treatments [68]. Notably, it is excelling in discovering biomarkers of promising new diagnostic methods for various illnesses. Through offering in-depth profiles and analysis data from MALDI-TOF MS, technology is not just beneficial for early detection purposes but also enables more accurate disease prognosis and monitoring capabilities to be established.

The application of MALDI-TOF MS technology in paleopathology opens new possibilities to study ancient diseases through different perspectives. The technology demonstrates its versatility through its ability to detect pathogens including *M. tuberculosis* and *P. falciparum* [98,102]. The examination of samples combined with historical disease trend analysis enables scientists to understand pathogen evolution while revealing their complex host-pathogen interactions across time. The example of plasmodium identification for paleopathological purposes before clinical purposes demonstrates how MALDI-TOF MS expansion across multiple fields accelerates its development [100]. Researchers from paleopathology and clinical microbiology who share common targets can achieve faster mutual discoveries through collaborative work. The field of comparative proteomic epidemiology shows great promise because it allows scientists to study evolutionary patterns by matching ancient pathogen spectra with modern equivalents [16]. The validation and optimization of protocols require essential collaboration between archaeological sciences and biomedical labs particularly for sample preparation and preservation assessment and database enrichment.

Although MALDI-TOF MS has become a gold standard in routine microbial identification and is increasingly implemented in clinical and research settings, it remains relatively underrecognized outside specialized laboratories when compared to other diagnostic technologies, such as PCR and next-generation sequencing [7]. This may be partly due to its relatively recent integration into clinical workflows, the requirement for specialized instrumentation and technical expertise, and the misconception that its applications are limited to microbiology [7]. Nevertheless, MALDI-TOF MS offers remarkable advantages, including rapid turnaround time, cost-effectiveness, and the ability to analyze a wide variety of biological samples with minimal preparation [113]. Importantly, it is not without its limitations. The performance of the technique is highly dependent on the quality and comprehensiveness of reference spectral databases, which currently lack sufficient representation of rare, fastidious, or unculturable organisms [113]. Moreover, the system still struggles to distinguish between closely related species and subspecies, as exemplified by the ongoing difficulty in differentiating *Shigella* species from *E. coli* [114,115]. Hygiene concerns and the risk of contamination may also be relevant in certain settings, although these are manageable through standardized cleaning protocols [116].

Despite these challenges, these limitations are not intrinsic to the technology and can be progressively addressed through continuous use, interdisciplinary collaboration, and expanded database curation. A key future direction lies in the enhancement of reference databases through the inclusion of high-quality spectra from diverse biological sources, contributed by a wide network of laboratories across multiple disciplines. In parallel, improvements in bioinformatics tools, including machine learning algorithms for pattern recognition, are expected to refine species- and strain-level discrimination. Furthermore, the potential of MALDI-TOF MS extends well beyond microbial diagnostics. Its versatility is increasingly demonstrated across diverse scientific domains such as paleopathology, environmental microbiology, aquaculture, oncology (e.g., detection of tumor-specific antigens), and biomarker discovery. These interdisciplinary applications underscore the need for strategic investment and broader recognition of MALDI-TOF MS as a central analytical platform. Its cost-effectiveness and high throughput make it especially attractive for use in resource-limited settings and large-scale screening programs.

MALDI-TOF MS is being positioned as a versatile analytical platform with transformative potential – even in areas such as paleopathology, where its application has been largely underexplored. By tracing the evolution of its capabilities and demonstrating its relevance across both contemporary and historical biomedical contexts, this review underscores MALDI-TOF MS as a unifying tool that can bridge diverse scientific disciplines. Through this lens, we encourage researchers and clinicians to consider its applicability not only within conventional boundaries but also in uncharted scientific territories where molecular analysis may provide critical insights. In doing so, we propose a broadened conceptual framework for how MALDI-TOF MS can inform future discovery and cross-disciplinary integration. To maximize its potential, MALDI-TOF MS should be adopted in parallel with established molecular techniques such as PCR, at least during the transition phase, to ensure diagnostic reliability and to facilitate the validation of its expanded use. As cumulative datasets grow and cross-disciplinary spectral overlap increases, the reliability and discriminatory power of the technique are expected to improve significantly.

Ultimately, the future of MALDI-TOF MS depends on sustained research, institutional support, and integration into routine workflows across various scientific and clinical domains. With targeted funding, enhanced inter-laboratory collaboration, and technological advancements, many of its current drawbacks can be overcome. This will not only strengthen its diagnostic utility but also unlock new avenues for research and innovation, making MALDI-TOF MS a cornerstone of precision diagnostics in the years to come.

## 8 Conclusions

MALDI-TOF MS has emerged as a transformative analytical platform with broad applicability across diverse scientific domains. Its ability to deliver rapid, accurate, and cost-effective molecular identifications has revolutionized clinical microbiology and is now gaining traction in fields previously considered outside its traditional scope, such as paleopathology. This review highlights the evolving capabilities of MALDI-TOF MS, tracing its development from a diagnostic tool in contemporary medicine to a key asset in historical biomolecular research. By demonstrating its relevance across both modern and ancient biomedical contexts, we position MALDI-TOF MS as a unifying technology that can bridge disciplinary divides. As advancements in AI integration, direct-from-sample processing, and bioinformatics continue to enhance its clinical utility, MALDI-TOF MS is also poised to redefine the analytical possibilities in paleoproteomics. However, realizing its full potential requires addressing current limitations, particularly the need for standardized spectral databases, improved taxonomic resolution, and refined protocols for handling minute or degraded samples. We advocate for a conceptual shift in how this technology is perceived – encouraging its adoption not only within established diagnostic frameworks but also in underexplored scientific frontiers. Thereby, MALDI-TOF MS may become a cornerstone for future discovery, innovation, and interdisciplinary integration.

## References

[1] Nakhod V, Krivenko A, Butkova T, Malsagova K, Kaysheva A. Advances in molecular and genetic technologies and the problems related to their application in personalized medicine. J Pers Med. 2024 May;14(6):555. 10.3390/jpm14060555 PMC11204801 38929775

[2] Franco-Duarte R, Černáková L, Kadam S, Kaushik KS, Salehi B, Bevilacqua A, et al. Advances in chemical and biological methods to identify microorganisms-from past to present. Microorganisms. 2019 Dec;7(5):130. 10.3390/microorganisms7050130 PMC6560418 31086084

[3] Swiner DJ, Jackson S, Burris BJ, Badu-Tawiah AK. Applications of mass spectrometry for clinical diagnostics: The influence of turnaround time. Anal Chem. 2020 Jan;92(1):183–202. 10.1021/acs.analchem.9b04901 PMC7896279 31671262

[4] Mojebi A, Wu P, Keeping S, Hale B, Chase JG, Beaubrun A. Clinical impact of rapid molecular diagnostic tests in patients presenting with viral respiratory symptoms: A systematic literature review. PLoS One. 2024;19(6):e0303560. 10.1371/journal.pone.0303560 PMC11175541 38870136

[5] Yates Iii RJ. A century of mass spectrometry: From atoms to proteomes. Nat Methods. 2011 Aug;8(8):633–7.

[6] Tőkés L. The allure of mass spectrometry: From an earlyday chemist’s perspective. Mass Spectrom Rev. 2017;36(4):520–42. 10.1002/mas.21499 PMC5484321 26999732

[7] Calderaro A, Chezzi C. MALDI-TOF MS: A reliable tool in the real life of the clinical microbiology laboratory. Microorganisms. 2024 Feb;12(2):322. 10.3390/microorganisms12020322 PMC10892259 38399726

[8] Tanaka K, Waki H, Ido Y, Akita S, Yoshida Y, Yoshida T, et al. Protein and polymer analyses up to m/z 100 000 by laser ionization time‐of‐flight mass spectrometry. Rapid Communications in Mass Spectrom. 1988;2:151–3. Wiley Online Library [Internet]. [cited 2025 May 8]. https://analyticalsciencejournals.onlinelibrary.wiley.com/doi/10.1002/rcm.1290020802.

[9] Fenselau C, Demirev PA. Characterization of intact microorganisms by maldi mass spectrometry. Mass Spectrom Rev. 2001;20(4):157–71. 10.1002/mas.10004 11835304

[10] Karas M, Hillenkamp F. Laser desorption ionization of proteins with molecular masses exceeding 10,000 daltons. Anal Chem. 1988 Oct;60(20):2299–301. 10.1021/ac00171a028 3239801

[11] DeLaney K, Phetsanthad A, Li L. Advances in high-resolution maldi mass spectrometry for neurobiology. Mass Spectrom Rev. 2022 Mar;41(2):194–214. 10.1002/mas.21661 PMC8106695 33165982

[12] Beck AG, Muhoberac M, Randolph CE, Beveridge CH, Wijewardhane PR, Kenttämaa HI, et al. Recent developments in machine learning for mass spectrometry. ACS Meas Sci Au. 2024 Jun;4(3):233–46. 10.1021/acsmeasuresciau.3c00060 PMC11191731 38910862

[13] Tarfeen N, Nisa KU, Nisa Q. MALDI-TOF MS: Application in diagnosis, dereplication, biomolecule profiling and microbial ecology. Proc Indian Natl Sci Acad Part Phys Sci. 2022;88(3):277–91.

[14] Cheon DH, Jang H, Choi YK, Oh WS, Hwang S, Park JR, et al. Clinical evaluation of advanced MALDI-TOF MS for carbapenemase subtyping in Gram-negative isolates. J Clin Microbiol. 2025 Jan;63(1):e0147524. 10.1128/jcm.01475-24 PMC11784181 39611795

[15] Hou TY, Chiang-Ni C, Teng SH. Current status of MALDI-TOF mass spectrometry in clinical microbiology. J Food Drug Anal. 2019 Apr;27(2):404–14. 10.1016/j.jfda.2019.01.001 PMC9296205 30987712

[16] Warinner C, Korzow Richter K, Collins MJ. Paleoproteomics. Chem Rev. 2022 Aug 24;122(16):13401–46. 10.1021/acs.chemrev.1c00703 PMC9412968 35839101

[17] Li D, Yi J, Han G, Qiao L. MALDI-TOF mass spectrometry in clinical analysis and research. ACS Meas Sci Au. 2022;2:385–404. [Internet]. [cited 2024 May 23]. https://pubs.acs.org/doi/full/10.1021/acsmeasuresciau.2c00019. 10.1021/acsmeasuresciau.2c00019 PMC9885950 36785658

[18] Smole SC, King LA, Leopold PE, Arbeit RD. Sample preparation of Gram-positive bacteria for identification by matrix assisted laser desorption/ionization time-of-flight. J Microbiol Methods. 2002 Feb;48(2):107–15. 10.1016/s0167-7012(01)00315-3 11777561

[19] Singhal N, Kumar M, Kanaujia PK, Virdi JS. MALDI-TOF mass spectrometry: An emerging technology for microbial identification and diagnosis. Front Microbiol. 2015 Aug;6:791. 10.3389/fmicb.2015.00791 PMC4525378 26300860

[20] Leopold J, Popkova Y, Engel KM, Schiller J. Recent developments of useful MALDI matrices for the mass spectrometric characterization of lipids. Biomolecules. 2018 Dec;8(4):173. 10.3390/biom8040173 PMC6316665 30551655

[21] Carbonnelle E, Grohs P, Jacquier H, Day N, Tenza S, Dewailly A, et al. Robustness of two MALDI-TOF mass spectrometry systems for bacterial identification. J Microbiol Methods. 2012 Dec;89(2):133–6. 10.1016/j.mimet.2012.03.003 22425492

[22] Sun J, Yu G, Yang Y, Qiao L, Xu B, Ding C, et al. Evaluation of prostate cancer based on MALDI-TOF MS fingerprinting of nanoparticle-treated serum proteins/peptides. Talanta. 2020 Dec;220:121331. 10.1016/j.talanta.2020.121331 32928383

[23] Friedrichs C, Rodloff AC, Chhatwal GS, Schellenberger W, Eschrich K. Rapid identification of viridans streptococci by mass spectrometric discrimination. J Clin Microbiol. 2007 Aug;45(8):2392–7. 10.1128/JCM.00556-07 PMC1951256 17553974

[24] Buckwalter SP, Olson SL, Connelly BJ, Lucas BC, Rodning AA, Walchak RC, et al. Evaluation of matrix-assisted laser desorption ionization-time of flight mass spectrometry for identification of Mycobacterium species, Nocardia species, and other aerobic Actinomycetes. J Clin Microbiol. 2016 Feb;54(2):376–84. 10.1128/JCM.02128-15 PMC4733196 26637381

[25] Alcalá L, Marín M, Ruiz A, Quiroga L, Zamora-Cintas M, Fernández-Chico MA, et al. Identifying anaerobic bacteria using MALDI-TOF mass spectrometry: A four-year experience. Front Cell Infect Microbiol. 2021 Apr;11:521014. 10.3389/fcimb.2021.521014 PMC8101409 33968791

[26] Almuzara M, Barberis C, Traglia G, Famiglietti A, Ramirez MS, Vay C. Evaluation of matrix-assisted laser desorption ionization-time-of-flight mass spectrometry for species identification of Nonfermenting Gram-Negative Bacilli. J Microbiol Methods. 2015 May;112:24–7. 10.1016/j.mimet.2015.03.004 25765149

[27] Jeong S, Hong JS, Kim JO, Kim KH, Lee W, Bae IK, et al. Identification of acinetobacter species using matrix-assisted laser desorption ionization-time of flight mass spectrometry. Ann Lab Med. 2016 Jul;36(4):325–34. 10.3343/alm.2016.36.4.325 PMC4855052 27139605

[28] Carbonnelle E, Beretti JL, Cottyn S, Quesne G, Berche P, Nassif X, et al. Rapid identification of Staphylococci isolated in clinical microbiology laboratories by matrix-assisted laser desorption ionization-time of flight mass spectrometry. J Clin Microbiol. 2007 Jul;45(7):2156–61. 10.1128/JCM.02405-06 PMC1932985 17507519

[29] Spanu T, De Carolis E, Fiori B, Sanguinetti M, D’Inzeo T, Fadda G, et al. Evaluation of matrix-assisted laser desorption ionization-time-of-flight mass spectrometry in comparison to rpoB gene sequencing for species identification of bloodstream infection staphylococcal isolates. Clin Microbiol Infect. 2011 Jan 1;17(1):44–9. 10.1111/j.1469-0691.2010.03181.x 20132252

[30] Sandu EC, Cursaru A, Iordache S, Serban B, Costache MA, Cirstoiu C. Utility of matrix-assisted laser desorption ionization time-of-flight mass spectrometry in periprosthetic joint infection diagnosis. Cureus. 2024 Oct;16(10):e70650. 10.7759/cureus.70650 PMC11527459 39483552

[31] Yamamoto K, Ono D, Nozaki Y, Nishida Y, Sakai J, Mimura K, et al. A case of Helicobacter cinaedi pleuritis and continuous ambulatory peritoneal dialysis-related peritonitis diagnosed by simultaneous-onset bacteremia. IDCases. 2025;40:e02197. 10.1016/j.idcr.2025.e02197 PMC11978351 40201413

[32] Mazhari F, Regberg AB, Castro CL, LaMontagne MG. Resolution of MALDI-TOF compared to whole genome sequencing for identification of Bacillus species isolated from cleanrooms at NASA Johnson Space Center. Front Microbiol. 2025;16:1499516. 10.3389/fmicb.2025.1499516 PMC12017291 40270816

[33] Nachtigall FM, Pereira A, Trofymchuk OS, Santos LS. Detection of SARS-CoV-2 in nasal swabs using MALDI-MS. Nat Biotechnol. 2020 Oct;38(10):1168–73. 10.1038/s41587-020-0644-7 32733106

[34] Yan L, Yi J, Huang C, Zhang J, Fu S, Li Z, et al. Rapid detection of COVID-19 using MALDI-TOF-based serum peptidome profiling. Anal Chem. 2021 Mar;93(11):4782–7. 10.1021/acs.analchem.0c04590 33656857

[35] Wang J, Zhang D, Chen Q, Yu J, Yang Y, Qiao L, et al. Establishment and application of PCR-time-of-flight mass spectrometer for SARS-CoV-2 nucleic acid detection. Chin Gen Pract. 2020 Dec;23(35):4430.

[36] Zhao Z, Sun L, Wang L, Li X, Peng J. A multiplex method for detection of SARS-CoV-2 variants based on MALDI-TOF mass spectrometry. Biosaf Health. 2023 Apr;5(2):101–7. 10.1016/j.bsheal.2023.02.003 PMC9977071 37123451

[37] Han Z, Yi J, Yang Y, Li D, Peng C, Long S, et al. SERS and MALDI-TOF MS based plasma exosome profiling for rapid detection of osteosarcoma. Analyst. 2021 Oct;146(21):6496–505. 10.1039/d1an01163d 34569564

[38] Rybicka M, Miłosz E, Bielawski KP. Superiority of MALDI-TOF mass spectrometry over real-time PCR for SARS-CoV-2 RNA detection. Viruses. 2021 May;13(5):730. 10.3390/v13050730 PMC8145549 33922195

[39] Costa MM, Martin H, Estellon B, Dupé FX, Saby F, Benoit N, et al. Exploratory study on application of MALDI-TOF-MS to detect SARS-CoV-2 infection in human saliva. J Clin Med. 2022 Jan;11(2):295. 10.3390/jcm11020295 PMC8781148 35053990

[40] Yegorov S, Kadyrova I, Korshukov I, Sultanbekova A, Kolesnikova Y, Barkhanskaya V, et al. Application of MALDI-TOF MS and machine learning for the detection of SARS-CoV-2 and non-SARS-CoV-2 respiratory infections. Microbiol Spectr. 2024 Dec;12(5):e0406823. 10.1128/spectrum.04068-23 PMC11064577 38497716

[41] Camarasa CG, Cobo F. Chapter twelve - Application of MALDI-TOF mass spectrometry in clinical virology. In: Cobo F, editor. The use of mass spectrometry technology (MALDI-TOF) in clinical microbiology. Cambridge, MA: Academic Press; 2018. p. 167–80. [cited 2024 Sep 22]. https://www.sciencedirect.com/science/article/pii/B9780128144510000125.

[42] Bader JC, Lakota EA, Flanagan S, Ong V, Sandison T, Rubino CM, et al. Overcoming the resistance hurdle: Pharmacokinetic-pharmacodynamic target attainment analyses for rezafungin (CD101) against Candida albicans and Candida glabrata. Antimicrob Agents Chemother. 2018 May;62(6):e02614–7. 10.1128/AAC.02614-17 PMC5971579 29555634

[43] Cassagne C, Normand AC, L’Ollivier C, Ranque S, Piarroux R. Performance of MALDI-TOF MS platforms for fungal identification. Mycoses. 2016 Nov;59(11):678–90. 10.1111/myc.12506 27061755

[44] Patel R. Matrix-assisted laser desorption ionization-time of flight mass spectrometry in clinical microbiology. Clin Infect Dis Publ Infect Dis Soc Am. 2013 Aug;57(4):564–72. 10.1093/cid/cit247 23595835

[45] Wieser A, Schneider L, Jung J, Schubert S. MALDI-TOF MS in microbiological diagnostics – identification of microorganisms and beyond (mini review). Appl Microbiol Biotechnol. 2012;93(3):965–74. 10.1007/s00253-011-3783-4 22198716

[46] Marklein G, Josten M, Klanke U, Müller E, Horré R, Maier T, et al. Matrix-assisted laser desorption ionization-time of flight mass spectrometry for fast and reliable identification of clinical yeast isolates. J Clin Microbiol. 2009 Sep;47(9):2912–7. 10.1128/JCM.00389-09 PMC2738125 19571014

[47] Bizzini A, Greub G. Matrix-assisted laser desorption ionization time-of-flight mass spectrometry, a revolution in clinical microbial identification. Clin Microbiol Infect Publ Eur Soc Clin Microbiol Infect Dis. 2010 Nov;16(11):1614–9. 10.1111/j.1469-0691.2010.03311.x 20636422

[48] van Veen SQ, Claas ECJ, Kuijper EJ. High-throughput identification of bacteria and yeast by matrix-assisted laser desorption ionization-time of flight mass spectrometry in conventional medical microbiology laboratories. 2010;48:900–7. PubMed [Internet]. [cited 2024 Sep 22] https://pubmed.ncbi.nlm.nih.gov/20053859/. 10.1128/JCM.02071-09 PMC2832429 20053859

[49] Normand AC, Cassagne C, Gautier M, Becker P, Ranque S, Hendrickx M, et al. Decision criteria for MALDI-TOF MS-based identification of filamentous fungi using commercial and in-house reference databases. BMC Microbiol. 2017 Jan;17(1):25. 10.1186/s12866-017-0937-2 PMC5282874 28143403

[50] Periyasamy M, Patel H, Lai CF, Nguyen VTM, Nevedomskaya E, Harrod A, et al. APOBEC3B-mediated cytidine deamination is required for estrogen receptor action in breast cancer. Cell Rep. 2015 Oct;13(1):108–21. 10.1016/j.celrep.2015.08.066 PMC4597099 26411678

[51] Swiatly A, Plewa S, Matysiak J, Kokot ZJ. Mass spectrometry-based proteomics techniques and their application in ovarian cancer research. J Ovarian Res. 2018 Oct;11:88. 10.1186/s13048-018-0460-6 PMC6166298 30270814

[52] Long S, Qin Q, Wang Y, Yang Y, Wang Y, Deng A, et al. Nanoporous silica coupled MALDI-TOF MS detection of Bence-Jones proteins in human urine for diagnosis of multiple myeloma. Talanta. 2019 Aug;200:288–92. 10.1016/j.talanta.2019.03.067 31036186

[53] Růžičková T, Vlachová M, Pečinka L, Brychtová M, Večeřa M, Radová L, et al. Detection of early relapse in multiple myeloma patients. Cell Div. 2025 Jan;20(1):4. 10.1186/s13008-025-00143-3 PMC11776158 39881385

[54] Zhou H, He X, Zhao J, Mei Z, Zhang X, Yuan W, et al. A MALDI-TOF mass spectrometry-based method for detection of copy number variations in BRCA1 and BRCA2 genes. Front Mol Biosci. 2023;10:1301652. 10.3389/fmolb.2023.1301652 PMC10808477 38274092

[55] Yan S, Zheng H, Zhao J, Gao M, Zhang X. Quantification of GPC1(+) exosomes based on MALDI-TOF MS in situ signal amplification for pancreatic cancer discrimination and evaluation. Anal Chem. 2023 Jul;95(27):10196–203. 10.1021/acs.analchem.3c00193 37368911

[56] Li Z, Chen J, Xu B, Zhao W, Zha H, Han Y, et al. Correlation between small-cell lung cancer serum protein/peptides determined by matrix-assisted laser desorption/ionization time-of-flight mass spectrometry and chemotherapy efficacy. Clin Proteom. 2024 Dec;21(1):35. 10.1186/s12014-024-09483-8 PMC11103996 38764042

[57] Fang E, Qian L, Tang J, Tong Z. Cytoplasmic expression of UTP23 promotes colorectal cancer progression. Cell Mol Biol. 2024 Jan;70(1):239–45. 10.14715/cmb/2024.70.1.33 38372088

[58] Zhu Y, Gasilova N, Jović M, Qiao L, Liu B, Tissières Lovey L, et al. Detection of antimicrobial resistance-associated proteins by titanium dioxide-facilitated intact bacteria mass spectrometry. Chem Sci. 2018;9(8):2212–21. 10.1039/c7sc04089j PMC5897883 29719694

[59] Ehrich M, Nelson MR, Stanssens P, Zabeau M, Liloglou T, Xinarianos G, et al. Quantitative high-throughput analysis of DNA methylation patterns by base-specific cleavage and mass spectrometry. Proc Natl Acad Sci U S A. 2005 Nov;102(44):15785–90. 10.1073/pnas.0507816102 PMC1276092 16243968

[60] Kim JI, Noh JY, Kim M, Park JM, Song HW, Kang MJ, et al. Newborn screening by matrix-assisted laser desorption/ionization mass spectrometry based on parylene-matrix chip. Anal Biochem. 2017 Aug;530:31–9. 10.1016/j.ab.2017.04.021 28465033

[61] Naubourg P, El Osta M, Rageot D, Grunewald O, Renom G, Ducoroy P, et al. A multicentre pilot study of a two-tier newborn sickle cell disease screening procedure with a first tier based on a fully automated MALDI-TOF MS platform. Int J Neonatal Screen. 2019 Mar;5(1):10. 10.3390/ijns5010010 PMC7510195 33072970

[62] El Osta M, Benoist JF, Naubourg P, Bonacorsi S, Messine R, Ducoroy P, et al. MALDI-MS in first-line screening of newborns for sickle cell disease: results from a prospective study in comparison to HPLC. Clin Chem Lab Med. 2024 Dec;62(6):1149–57. 10.1515/cclm-2023-1250 38353144

[63] Huang L, Zhang Q, Ye Y, Long Y, Huang H, Niu C, et al. Rapid detection of genetic modifiers of β-thalassemia based on MALDI-TOF MS. Ann Hematol. 2025 Mar;104(3):1481–92. 10.1007/s00277-025-06277-2 PMC12031963 40016399

[64] Renoux C, Roland E, Ruet S, Zouaghi S, Michel M, Joly P, et al. Evaluation of a new tandem mass spectrometry method for sickle cell disease newborn screening. Int J Neonatal Screen. 2024 Nov;10(4):77. 10.3390/ijns10040077 PMC11676960 39728397

[65] Meng Q, Ge S, Yan W, Li R, Dou J, Wang H, et al. Screening for potential serum-based proteomic biomarkers for human type 2 diabetes mellitus using MALDI-TOF MS. Proteom – Clin Appl. 2017;11(3–4):1600079. 10.1002/prca.201600079 27863080

[66] Wan M, Wang Y, Zhan L, Fan J, Hu TY. MALDI-TOF mass spectrometry-based quantification of C-peptide in diabetes patients. Eur J Mass Spectrom. 2019;25(4):345–50. [cited 2024 Sep 16] https://journals.sagepub.com/doi/abs/10.1177/1469066719865265. 10.1177/1469066719865265 PMC8104466 31319703

[67] Zawada A, Naskręt D, Matuszewska E, Kokot Z, Grzymisławski M, Zozulińska-Ziółkiewicz D, et al. MALDI-TOF protein profiling reflects changes in type 1 diabetes patients depending on the increased amount of adipose tissue, poor control of diabetes and the presence of chronic complications. Int J Environ Res Public Health. 2021 Jan;18(5):2263. 10.3390/ijerph18052263 PMC7967698 33668851

[68] Sánchez-Valencia PE, Díaz-García JD, Leyva-Leyva M, Sánchez-Aguillón F, González-Arenas NR, Mendoza-García JG, et al. Frequency of tumor necrosis factor-α, interleukin-6, and interleukin-10 gene polymorphisms in Mexican patients with diabetic retinopathy and diabetic kidney disease. Pathophysiol J Int Soc Pathophysiol. 2025 Apr;32(2):14. 10.3390/pathophysiology32020014 PMC12015769 40265439

[69] Jiang M, Li W, Wang D, Wu X, Chen D, Feng Y. Identification and investigation of protein-related molecules in patients with hyperlipidemia using label-free combined with bioinformatics analysis. Cell Mol Biol. 2023 Dec;69(13):262–9. 10.14715/cmb/2023.69.13.39 38158657

[70] Grela A, Turek A, Piekoszewski W. Application of matrix-assisted laser desorption/ionization time-of-flight mass spectrometry (MALDI-TOF MS) in Alzheimer’s disease. Clin Chem Lab Med. 2012 Feb;50(8):1297–304. 10.1515/cclm-2011-0550 22868793

[71] Vigo-Pelfrey C, Lee D, Keim P, Lieberburg I, Schenk DB. Characterization of beta-amyloid peptide from human cerebrospinal fluid. J Neurochem. 1993 Nov;61(5):1965–8. 10.1111/j.1471-4159.1993.tb09841.x 8229004

[72] Nakamura A, Kaneko N, Villemagne VL, Kato T, Doecke J, Doré V, et al. High performance plasma amyloid-β biomarkers for Alzheimer’s disease. Nature. 2018 Feb;554(7691):249–54. 10.1038/nature25456 29420472

[73] Xing X, Ji X, Liu X, Jin X, He Z, Xu A, et al. Development and validation of a one-step SMN assay for genetic testing in spinal muscular atrophy via MALDI-TOF MS. Analyst. 2024 Dec;150(1):142–53. 10.1039/d4an01225a 39584662

[74] Deulofeu M, Peña-Méndez EM, Vaňhara P, Havel J, Moráň L, Pečinka L, et al. Discriminating fingerprints of chronic neuropathic pain following spinal cord injury using artificial neural networks and mass spectrometry analysis of female mice serum. Neurochem Int. 2024 Dec;181:105890. 10.1016/j.neuint.2024.105890 39455011

[75] Zhao C, Sun Z, Wang S, Zhang J, Liu J, Chen L, et al. IgG4 glycosylation contributes to the pathogenesis of IgG4 Hashimoto’s thyroiditis via the complement pathway. Eur Thyroid J. 2024 Oct;13(5):e240156. 10.1530/ETJ-24-0156 PMC11558973 39316722

[76] Gao S, Jia M, Wang J, Sun Q, Liu F, Yu L, et al. Association of ADAMTS-5 gene polymorphisms with the susceptibility to knee osteoarthritis in a Chinese Han population. J Orthop Surg. 2024 Aug;19(1):513. 10.1186/s13018-024-05023-0 PMC11348706 39192347

[77] Oviaño M, Rodríguez-Sánchez B, Gómara M, Alcalá L, Zvezdanova E, Ruíz A, et al. Direct identification of clinical pathogens from liquid culture media by MALDI-TOF MS analysis. Clin Microbiol Infect Publ Eur Soc Clin Microbiol Infect Dis. 2018 Jun;24(6):624–9. 10.1016/j.cmi.2017.09.010 28962998

[78] Schuster D, Josten M, Janssen K, Bodenstein I, Albert C, Schallenberg A, et al. Detection of methicillin-resistant coagulase-negative staphylococci harboring the class A mec complex by MALDI-TOF mass spectrometry. Int J Med Microbiol IJMM. 2018 Jul;308(5):522–6. 10.1016/j.ijmm.2018.05.001 29764754

[79] Rodríguez-Sánchez B, Cercenado E, Coste AT, Greub G. Review of the impact of MALDI-TOF MS in public health and hospital hygiene, 2018. Euro Surveill Bull Eur Sur Mal Transm Eur Commun Dis Bull. 2019 Jan;24(4):1800193. 10.2807/1560-7917.ES.2019.24.4.1800193 PMC6351997 30696525

[80] Nwabufo CK, Aigbogun OP. Diagnostic and therapeutic agents that target alpha-synuclein in Parkinson’s disease. J Neurol. 2022;269(11):5762–86. 10.1007/s00415-022-11267-9 PMC9281355 35831620

[81] Swales JG, Tucker JW, Strittmatter N, Nilsson A, Cobice D, Clench MR, et al. Mass spectrometry imaging of cassette-dosed drugs for higher throughput pharmacokinetic and biodistribution analysis. Anal Chem. 2014 Aug;86(16):8473–80. 10.1021/ac502217r 25084360

[82] Herkt M, Foinquinos A, Batkai S, Thum T, Pich A. Pharmacokinetic studies of antisense oligonucleotides using MALDI-TOF mass spectrometry. Front Pharmacol. 2020 Mar;11:220. [cited 2024 Sep 16] https://www.frontiersin.org/journals/pharmacology/articles/10.3389/fphar.2020.00220/full. 10.3389/fphar.2020.00220 PMC7109322 32269522

[83] Zhang N, Baran A, Valioglu F, Teng L, Atalar MN, Keskin C, et al. Antioxidant, AChE inhibitory, and anticancer effects of Verbascum thapsus extract. Cell Mol Biol. 2023 Dec;69(14):211–6. 10.14715/cmb/2023.69.14.35 38279434

[84] Xu W, Qian J, Xu Q, Cai R, Yang G, Wei J, et al. Proteins and signaling pathways response to Wenjingtongluo drug-contained serum in IHUVECs: An explorative proteomic study. Cell Mol Biol. 2023 Jun;69(6):116–24. 10.14715/cmb/2023.69.6.18 37605582

[85] Hamidi H, Bagheri Nejad R, Es-Haghi A, Ghassempour A. A combination of MALDI-TOF MS proteomics and species-unique biomarkers’ discovery for rapid screening of brucellosis. J Am Soc Mass Spectrom. 2022;33(10):1530–9. [cited 2024 Sep 22] https://pubs.acs.org/doi/abs/10.1021/jasms.2c00110. 10.1021/jasms.2c00110 35816556

[86] Vestal M, Hayden K. High performance MALDI-TOF mass spectrometry for proteomics. Int J Mass Spectrom. 2007 Dec;268(2–3):83–92.

[87] Spraggins JM, Rizzo DG, Moore JL, Noto MJ, Skaar EP, Caprioli RM. Next-generation technologies for spatial proteomics: Integrating ultra-high speed MALDI-TOF and high mass resolution MALDI FTICR imaging mass spectrometry for protein analysis. Proteomics. 2016 Jun;16(11–12):1678–89. 10.1002/pmic.201600003 PMC5117945 27060368

[88] Abreu R, Matos A, Capela L, Jorge R, Guerreiro JF, Pereira G, et al. Extended-spectrum β-lactamase-producing Klebsiella pneumoniae in dogs from Cape Verde and São Tomé and Príncipe: Implications for public health. Antibiotics Basel Switz. 2025 Apr 16;14(4):408. 10.3390/antibiotics14040408 PMC12023937 40298570

[89] Jiwaganont P, Roytrakul S, Thaisakun S, Sukumolanan P, Petchdee S. Investigation of coagulation and proteomics profiles in symptomatic feline hypertrophic cardiomyopathy and healthy control cats. BMC Vet Res. 2024 Jul 5;20(1):292. 10.1186/s12917-024-04170-0 PMC11225243 38970022

[90] Hisirová S, Koščová J, Király J, Hajdučková V, Hudecová P, Lauko S, et al. Resistance genes and virulence factor genes in coagulase-negative and positive staphylococci of the staphylococcus intermedius Group (SIG) isolated from the dog skin. Microorganisms. 2025 Mar;13(4):735. 10.3390/microorganisms13040735 PMC12029769 40284572

[91] Becker P, Normand AC, Vanantwerpen G, Vanrobaeys M, Haesendonck R, Vercammen F, et al. Identification of fungal isolates by MALDI-TOF mass spectrometry in veterinary practice: Validation of a web application. J Vet Diagn Investig Publ Am Assoc Vet Lab Diagn Inc. 2019 Dec;31(3):471–4. 10.1177/1040638719835577 PMC6838695 30943879

[92] Hollemeyer K, Altmeyer W, Heinzle E, Pitra C. Species identification of Oetzi’s clothing with matrix-assisted laser desorption/ionization time-of-flight mass spectrometry based on peptide pattern similarities of hair digests. Rapid Commun Mass Spectrom RCM. 2008 Sep;22(18):2751–67. 10.1002/rcm.3679 18720427

[93] Mark L, Zoltán P, Vaczy A, Lorand T, Marcsik A. High-throughput mass spectrometric analysis of 1400-year-old mycolic acids as biomarkers for ancient tuberculosis infection. J Archaeol Sci. 2010 Feb;37:302–5.

[94] Boros-Major A, Bona A, Lovász G, Molnar E, Marcsik A, Pálfi G, et al. New perspectives in biomolecular paleopathology of ancient tuberculosis: A proteomic approach. J Archaeol Sci. 2011 Jan;38:197–201.

[95] Hajdu T, Fóthi E, Kovári I, Merczi M, Molnár A, Maász G, et al. Bone tuberculosis in Roman Period Pannonia (western Hungary). Mem Inst Oswaldo Cruz. 2012 Dec;107(8):1048–53. 10.1590/s0074-02762012000800014 23295757

[96] Taylor G, Blau S, Mays S, Monot M, Lee O, Minnikin D, et al. Mycobacterium leprae genotype amplified from an archaeological case of lepromatous leprosy in Central Asia. J Archaeol Sci. 2009 Oct;36(10):2408–14.

[97] Stauning MA, Jensen CS, Staalsøe T, Kurtzhals JAL. Detection and quantification of Plasmodium falciparum in human blood by matrix-assisted laser desorption/ionization time-of-flight mass spectrometry: A proof of concept study. Malar J. 2023 Sep 26;22(1):285. 10.1186/s12936-023-04719-8 PMC10523782 37752504

[98] Inwood J. Identifying malaria in ancient human remains. A molecular and biochemical approach [Doctoral dissertation]. New Haven, CT: Yale University; 2017.

[99] Ayyadurai S, Flaudrops C, Raoult D, Drancourt M. Rapid identification and typing of Yersinia pestis and other Yersinia species by matrix-assisted laser desorption/ionization time-of-flight (MALDI-TOF) mass spectrometry. BMC Microbiol. 2010 Nov;10(1):285. 10.1186/1471-2180-10-285 PMC2992509 21073689

[100] Mai B, Drancourt M, Aboudharam G. Ancient dental pulp: Masterpiece tissue for paleomicrobiology. Mol Genet Genomic Med. 2020 Mar;8:e1202. 10.1002/mgg3.1202 PMC7284042 32233019

[101] Appelt S, Armougom F, Bailly ML, Robert C, Drancourt M. Polyphasic analysis of a middle ages coprolite microbiota, Belgium. PLoS One. 2014 Feb;9(2):e88376. 10.1371/journal.pone.0088376 PMC3938422 24586319

[102] Bona Á, Papai Z, Maasz G, Toth GA, Jambor E, Schmidt J, et al. Mass spectrometric identification of ancient proteins as potential molecular biomarkers for a 2000-year-old osteogenic sarcoma. PLoS One. 2014;9(1):e87215. 10.1371/journal.pone.0087215. PMC3903643 24475253

[103] Çağatay İT. Use of proteomic-based MALDI-TOF mass spectra for identification of bacterial pathogens in aquaculture: A review. Aquac Int. 2024 Dec;32(6):7835–71.

[104] Zwies C, Vargas Rodríguez ÁM, Naumann M, Seifert F, Pietzsch M. Alternative strategies for the recombinant synthesis, DOPA modification and analysis of mussel foot proteins - A case study for Mefp-3 from Mytilus edulis. Protein Expr Purif. 2024 Jul;219:106483. 10.1016/j.pep.2024.106483 38609025

[105] De Pascale S, Garro G, Pellicano SI, Scaloni A, Carpino S, Caira S, et al. Integrated gel electrophoresis and mass spectrometry approach for detecting and quantifying extraneous milk in protected designation of origin Buffalo Mozzarella Cheese. Foods Basel Switz. 2025 Mar 28;14(7):1193. 10.3390/foods14071193 PMC11988604 40238338

[106] Augustyńska-Prejsnar A, Kačániová M, Hanus P, Sokołowicz Z, Słowiński M. Microbial and sensory quality changes in broiler chicken breast meat during refrigerated storage. Foods. 2024;13:4063. [Internet]. [cited 2025 May 12]. https://www.mdpi.com/2304-8158/13/24/4063. 10.3390/foods13244063 PMC11675927 39767005

[107] Tamura H. Bacterial pesticides: Mechanism of action, possibility of food contamination, and residue analysis using MS. J Pestic Sci. 2024;49(3):135–47. 10.1584/jpestics.D24-006 PMC11464265 39398503

[108] Duncan M, DeMarco ML. MALDI-MS: Emerging roles in pathology and laboratory medicine. Clin Mass Spectrom. 2019 May;13:1–4. 10.1016/j.clinms.2019.05.003 PMC8620520 34841079

[109] Seng P, Abat C, Rolain JM, Colson P, Lagier JC, Gouriet F, et al. Identification of rare pathogenic bacteria in a clinical microbiology laboratory: impact of matrix-assisted laser desorption ionization-time of flight mass spectrometry. J Clin Microbiol. 2013 Jul;51(7):2182–94. 10.1128/JCM.00492-13 PMC3697718 23637301

[110] van Belkum A, Chatellier S, Girard V, Pincus D, Deol P, Dunne WM. Progress in proteomics for clinical microbiology: MALDI-TOF MS for microbial species identification and more. Expert Rev Proteom. 2015;12(6):595–605. 10.1586/14789450.2015.1091731 26472137

[111] Vrioni G, Tsiamis C, Oikonomidis G, Theodoridou K, Kapsimali V, Tsakris A. MALDI-TOF mass spectrometry technology for detecting biomarkers of antimicrobial resistance: current achievements and future perspectives. Ann Transl Med. 2018 Jun;6(12):240. 10.21037/atm.2018.06.28 PMC6046294 30069442

[112] Yamin D, Uskoković V, Wakil AM, Goni MD, Shamsuddin SH, Mustafa FH, et al. Current and future technologies for the detection of antibiotic-resistant bacteria. Diagnostics. 2023 Oct;13(20):3246. 10.3390/diagnostics13203246 PMC10606640 37892067

[113] Rychert J. Benefits and limitations of MALDI-TOF mass spectrometry for the identification of microorganisms. J Infect Epidemiol. 2019 Feb;2(4):1–5. [cited 2025 May 11] https://www.infectiologyjournal.com/articles/benefits-and-limitations-of-malditof-mass-spectrometry-for-the-identification-of-microorganisms.html?utm_source=chatgpt.com.

[114] van den Beld MJC, Rossen JWA, Evers N, Kooistra-Smid MAMD, Reubsaet FAG. MALDI-TOF MS using a custom-made database, biomarker assignment, or mathematical classifiers does not differentiate Shigella spp. and Escherichia coli. Microorganisms. 2022 Feb;10(2):435. 10.3390/microorganisms10020435 PMC8878589 35208889

[115] Martiny D, Busson L, Wybo I, El Haj RA, Dediste A, Vandenberg O. Comparison of the Microflex LT and Vitek MS systems for routine identification of bacteria by matrix-assisted laser desorption ionization–time of flight mass spectrometry. J Clin Microbiol. 2012 Apr;50(4):1313–25. 10.1128/JCM.05971-11 PMC3318529 22322345

[116] Cleaning procedure for MALDI sample targets [Internet]. [cited 2025 May 11]. https://www.ssi.shimadzu.com/service-support/technical-support/handling-precautions/maldi/fleximass/index.html.

